# Alcohol consumption of adults in Germany: Harmful drinking quantities, consequences and measures

**DOI:** 10.17886/RKI-GBE-2016-029

**Published:** 2016-09-28

**Authors:** Cornelia Lange, Kristin Manz, Alexander Rommel, Anja Schienkiewitz, Gert B. M. Mensink

**Affiliations:** Robert Koch Institute, Department for Epidemiology and Health Monitoring, Berlin, Germany

**Keywords:** HARMFUL ALCOHOL CONSUMPTION, ALCOHOL ABUSE, ADULTS, HEALTH SURVEY, TRENDS OVER TIME

## Abstract

Harmful alcohol consumption is one of the five essential risk factors for disease, impairments and premature death around the world. It is considered to be a contributory cause for more than 200 diseases and is co-responsible for causing many intentional and unintentional injuries.

In order to reduce harmful alcohol consumption, the health target “Reduce alcohol consumption” has been currently elaborated in Germany and focuses on a policy mix of behavioural and situational preventive measures to include as far as possible all relevant players for the development of overarching objectives.

The data from the recurrent health surveys by the Robert Koch Institute (RKI) allow an evaluation of trends of harmful alcohol consumption in the population aged 25 to 69 between 1990/1992, 1997/1999, and 2008/2011. Harmful alcohol consumption is defined as a daily consumption of pure alcohol of more than 10g for women and more than 20g for men. For the years 2008-2011 harmful alcohol consumption for the age group 18 to 79 years is calculated based on the “German Health Interview and Examination Survey for Adults” (DEGS1) and examined in connection with socio-demographic and health-related factors.

The results of DEGS1 show that 13.1% of women and 18.5% of men consume alcohol in harmful quantities. For men harmful alcohol consumption rises with the age; for women the lowest prevalence is found in those aged 30-39 years and the highest in the age group 50-59 years. Women with a high socio-economic status drink a harmful quantity of alcohol to a higher extent than women from medium or low status groups. For men there are no corresponding differences. Mainly smoking is associated with harmful alcohol consumption. Between 1990 and 1992 as well as between 2008 and 2011 harmful alcohol consumption has strongly declined, for women from 50.9% to 13.6%, for men from 52.6% to 18.3% (age group 25 to 69 years). Even if harmful alcohol consumption in the population has strongly declined, the per capita consumption of pure alcohol is above the average of the EU Member States in Germany. For that reason, preventive measures for specific target groups are required.

## 1. Introduction

### 1.1 Health and social consequences of harmful alcohol consumption

**Infobox: Exclusively alcohol-related diseases [2]**

**Table:** 

*ICD-10*	*Explanation*
E24.4	Alcohol-induced Pseudo-Cushing Syndrome
E52	Niacin deficiency (pellagra)
F10	Mental and behavioural disorders due to use of alcohol
G31.2	Degeneration of nervous system due to alcohol
G62.1	Alcoholic polyneuropathy
G72.1	Alcoholic myopathy
I42.6	Alcoholic cardiomyopathy
K29.2	Alcoholic gastritis
K70	Alcoholic liver disease
K85.2	Alcohol-induced acute pancreatitis (from 2006)
K86.0	Alcohol-induced chronic pancreatitis
O35.4	Maternal care for (suspected) damage to foetus from alcohol
P04.3	Foetus and new-born affected by maternal use of alcohol
Q86.0	Foetal alcohol syndrome (dysmorphic)
R78.0	Finding of alcohol in blood
T51.0	Toxic effect: ethanol
T51.9	Toxic effect: alcohol, unspecified

The consumption of alcoholic beverages has been spread in many cultures for a very long time. In traditional societies alcoholic beverages were prepared artisanally in small quantities and mainly consumed on special occasions such as celebrations. With industrialisation, the production and availability of alcohol changed. Spirits were introduced and as a result of improved production and transport conditions alcoholic beverages became a product which was available at any time of the year and every day of the week. Since the industrialised societies needed sober and attentive workers, excessive alcohol consumption was considered during the late 19th century as a growing social problem and burden for public health. As a result, increasing measures were taken to reduce or prohibit drinking [1].

Alcohol is a psychoactive substance which can cause dependence. Harmful alcohol consumption is, moreover, one of the five essential risk factors for diseases, impairments and premature death around the world. It is considered to be a contributory cause for more than 200 diseases and is co-responsible for causing many intentional and unintentional injuries [1].

The German Federal Statistical Office has compiled together with the German Institute for Medical Documentation and Information (DIMDI) a list of 17 diseases which are to be considered 100% as alcohol-related (cf. Infobox Exclusively alcohol-related diseases) [2]. According to this list, for 14,099 deceased in Germany in 2014 an exclusively alcohol-related disease was main cause of death [3].

Estimates from the “Global Burden of Disease Study” show, moreover, that on a worldwide level 5% of all disability-adjusted life years (DALYs) can be attributed to alcohol [4]. In Germany alcohol consumption ranges amongst all risk factors for DALYs in men fifth and in women eighth, referred to the year 2013 [5].

As far as the effects of alcohol consumption are concerned, the World Health Organisation (WHO) distinguishes not only between health consequences for consumers but also between socio-economic consequences for those concerned as well as damage to other persons and the society as a whole.

Damage for the individual includes chronic damage to tissues and organs due to the toxic effect of alcohol (harmful use or abuse, ICD-10: F10.1), acute alcohol intoxication which can consist in disturbances of co-ordination, consciousness, cognition and perception (ICD10: F10.0), as well as the development of an alcohol dependence (dependence syndrome ICD-10: F10.2).

The individual socio-economic consequences of harmful, abusive or dependent alcohol consumption can reach from stigmatisation, social withdrawal, family problems to the loss of job, residence and complete social exclusion. Damages for third parties are mainly caused by bodily injuries following violence or accidents, mental injuries and strains of partners, family, friends, colleagues as well as damages to unborn children (fetal alcohol spectrum disorder – FASD).

The social consequences of alcohol consumption include, apart from the direct costs for the health system, productivity losses as well as absenteeism at the workplace or early retirements as well as intangible costs, for instance as a result of a loss of quality of life. These economic costs of alcohol consumption in Germany are assessed according to estimates to reach an amount of up to EUR 40 billion per year; approximately one-third is accounted for by direct costs for the health system [2, 6].

### 1.2 Health policy initiatives to reduce alcohol consumption

On the international, European and national level there are a series of initiatives and strategies to reduce alcohol consumption in the population [7]. In the WHO “Global action plan for the prevention and control of non-communicable diseases” a relative reduction of the risk alcohol consumption by 10% until 2025 (versus 2010) is striven for. The objectives of the global WHO strategy to reduce harmful use of alcohol are:

► raise global awareness of the magnitude and nature of the health, social and economic problems caused by harmful use of alcohol, and increased commitment by governments to act to address harmful use of alcohol;
► strengthened knowledge base on the magnitude and determinants of alcohol-related harm and on effective interventions to reduce and prevent such harm;
► increased technical support to, and enhanced capacity of, Member States for preventing the harmful use of alcohol and managing alcohol-use disorders and associated health conditions;
► strengthen partnerships and better co-ordination among stakeholders and increased mobilisation of resources required for appropriate and concerted action to prevent the harmful use of alcohol;
► improved systems for monitoring and surveillance.

In detail, 10 fields of action are mentioned in which governments should act. They are: (1) Leadership, awareness and commitment, (2) Health services’ response, (3) Community action, (4) Drink-driving policies and counter measures, (5) Availability of alcohol, (6) Marketing of alcoholic beverages, (7) Pricing policies, (8) Reducing the negative consequences of drinking and alcohol intoxication, (9) Reducing the public health impact of illicit alcohol and informally produced alcohol and (10) Monitoring and surveillance. For each of these fields of action, action strategies have been formulated and priority fields of action are referred to [8]. In the “European action plan to reduce the harmful use of alcohol 2012-2020” of WHO [7] the fields of action of the global strategy are referred to and adapted to the European Region of WHO.

The European Commission submitted a strategy to support Member States in reducing alcohol-related harm in 2006 [9]. Since regulations in the field of health come within the sphere of responsibility of the Member States, it does not have a binding character. It focuses on the prevention of high and extreme alcohol consumption as well as the reduction of alcohol consumption of young people and some of the most negative effects of alcohol-related traffic accidents and the fetal alcohol syndrome. The strategy is hence not dealing with alcohol consumption as such but with its abuse and its damaging effects. Five areas were mentioned in which a joint approach of the Member States can have an added value:

► protect young people, children and the unborn child,
► reduce injuries and death from alcohol-related road-accidents;
► prevent alcohol-related harm among adults and reduce the negative impact on the workplace;
► inform, educate and raise awareness on the impact of harmful and harmful alcohol consumption, and on appropriate consumption patterns;
► develop and maintain a common evidence base at EU level.

On a national level, the national strategy for drug and addiction policy is guiding the different actions [10]. It stresses that a successful alcohol prevention requires a bundle of regulations, information and behavioural preventive measures, and alcohol prevention must be seen as a social cross-sectional task (“policy mix”). The national strategy refers to eight goals to fight alcohol consumption and its consequences:

► the reduction of the frequency of heavy episodic drinking amongst children and adolescents;
► the consistent implementation of the existing regu lations of the Youth Protection Act;
► the protection of children and adolescents from alcohol advertising;
► the reduction of alcohol consumption in road traffic;
► the situational abstinence at the workplace;
► the situational abstinence during pregnancy and breastfeeding;
► the reduction of alcohol-related violence;
► the concentration on risk groups in the adult population.

As far as advertising for alcoholic beverages is concerned, the focus is on the self-control of the alcohol industry which is to be evaluated by an independent body. In order to achieve situational abstinence at the workplace, the strategy relies on the promotion of corporate agreements as well as model projects on addiction prevention at the workplace. As far as the fields of action of pricing and availability of alcohol are concerned, the strategy does not include any statements. Concerning the availability of alcohol, there are, however, some provisions in the Youth Protection Act as well as in the Restaurant and Pub Act. Some federal states or municipalities have adopted regulations on the selling time for alcohol, restrictions of consumption through substantiated local prohibitions of alcohol or alcohol prohibitions in public transportation. Overall, there are to a large extent no statutory provisions in the field of pricing, advertising and availability. Germany is amongst the European countries in which a comparatively high alcohol consumption goes along with low statutory restrictions [11]. The health target “Reduce alcohol consumption”, which was published in a first version in 2015, the fields of action advertising, pricing and availability are still to be tackled and corresponding goals are to be elaborated [12].

**Infobox: Forms of alcohol consumption harmful to health**

**Harmful alcohol consumption**

A consumption pattern is designated as consumption of harmful drinking amounts of alcohol if the risk of damaging consequences for the bodily and mental health are increased [47]. An average daily alcohol drinking amount of more than 10-12g for women and 20-24g pure alcohol for men is defined as harmful [45, 48].

**Heavy episodic drinking**

Heavy episodic drinking (HED) refers to the consumption of 60g or more of pure alcohol at a drinking occasion taking place at least once a month. This amount corresponds to the consumption of six standard glasses of alcoholic beverages which contain approximately 10g of pure alcohol per glass each.

**Alcohol abuse (harmful alcohol consumption, damaging use)**

Alcohol abuse designates a consumption pattern which leads to physical and mental health damages and normally goes along with a habitual consumption of large amounts of alcohol. According to ICD-10 alcohol abuse (ICD-10: F10.1) is differentiated from alcohol dependence (ICD-10: F10.2) insofar as abuse does not (yet) involve an overwhelming craving or compulsion to consume [49].

**Alcohol dependency**

Alcohol dependency exists if there is a strong and frequently not controllable craving to consume alcohol. At the same time there is a metal concentration on alcohol consumption and a loss of control over the drinking quantity [49].

**Alcohol use disorder**

An alcohol use disorder exists in accordance with DSM-5 if a person meets certain diagnostic criteria. These include, for instance, difficulties to control alcohol consumption, the continuation of consumption despite problems resulting from the alcohol consumption, a habituation and withdrawal symptoms and the lasting craving for alcohol. The overlapping in terms of content with the diagnostic criteria for alcohol abuse and alcohol dependence is due to the fact that upon the introduction of DSM-5, abuse and dependence were merged in a diagnostic system for the joint diagnosis of alcohol use disorder.

The practical implementation of measures for alcohol prevention is based on a series of projects and campaigns. These include, more particularly, the activities the Federal Centre for Health Education (BZgA), for instance with the campaign “Know your limit” for adults and adolescents and a campaign “Zero alcohol – full power” for the target group of the 12-16-year-olds as well as the national “Alcohol Action Week” of the German Centre for Addiction Issues (cf. [2]). The alcohol prevention project “HaLT” (“Close to the limit”) combines approaches on an individual and municipal level and targets, more particularly, young people who have already attracted attention because of harmful alcohol consumption.

In order to verify how the consumption of alcoholic beverages and its health and social effects develop, continuous monitoring is necessary. In the long-term this represents an important aspect to review the achievement of goals of initiatives such as the health target “Reduce alcohol consumption” as well as the efficacy of the general social efforts through situational and behavioural preventive measures.

The goal of this paper is to present, based on the data of health monitoring at the Robert Koch Institute, the prevalence of the consumption of harmful alcohol drinking quantities in the adult population in Germany and to identify the relationship with important socio-demographic and health-related factors. Since the adult review surveys – the East/West survey 1991 (OW91), the Federal Health Survey 1998 (BGS98) as well as the study on the health of adults in Germany (DEGS1) – permit the representation of the development over the past 25 years, the long-term development of the harmful alcohol consumption shall be analyzed. Consequently, these results represent an important supplement to the already existing trend analyses [13-15].

## 2. Method

### 2.1 Measurement of alcohol consumption in the population – data sources and indicators

In general, science distinguishes between different forms of alcohol consumption with a hazard to health and its consequences. These include the consumption of harmful alcohol drinking quantities, heavy episodic drinking, alcohol abuse, alcohol dependence and alcohol use disorder (see Infobox Forms of alcohol consumption harmful to health). Within the framework of its “Global action plan for the prevention and control of non-communicable diseases” [16] WHO specifies as a minimal set of indicators 1. Per capita consumption of pure alcohol for consumers aged ≥15, 2. Age-standardised prevalence of heavy episodic drinking among adolescents and adults and 3. Alcohol-related morbidity and mortality among adolescents and adults. In addition, WHO points out that these indicators may be put together in accordance with the national context and may be supplemented by additional indicators.

This study concentrates on the prevalence of harmful alcohol consumption. Supplementary information on the per capita consumption of pure alcohol can be found in the discussion section. Moreover, the fact sheets include information on the topics alcohol-related mortality, road traffic accidents under the influence of alcohol and acute alcohol intoxications with in-patient treatment.

In order to determine the alcohol consumption in the population, there is on the one hand the possibility to rely on data from excise duty statistics and on the other hand interview data from representative studies can be used. A significant predictor for alcohol-related negative health and social consequences is the per capita consumption of alcoholic beverages [2]. Representative population surveys offer the opportunity to describe different drinking patterns in the population in detail and analyse them by socio-demographic characteristics such as age and gender. The alcohol consumption is determined in specific population studies through special survey instruments such as the Alcohol Use Disorder Identification Test (-Consumption) (AUDIT and AUDIT-C) [17] or frequency quantity indexes which determine the frequency and quantity of consumption of specific alcoholic beverages which are converted to an average consumption of pure alcohol in gram per day. Moreover, there are specific tools to survey substance-related disorders (here: alcohol abuse and alcohol dependency) such as the Munich Composite International Diagnostic Interview (M-CIDI; [18]).

### 2.2 Included studies

DEGS1 is an integral part of the health monitoring by the Robert Koch Institute. The study design and goals of DEGS1 have been described in detail elsewhere [19, 20]. DEGS1 was carried out between 2008 and 2011. The target population was the residential population living in Germany aged between 18 and 79 years. DEGS1 has a mixed design which permits both cross-sectional and longitudinal analyses. In this connection registry office samples were supplemented by former participants in BGS98. Altogether 8,151 persons participated, including 4,192 persons invited for the first time (response rate 42%) and 3,959 former BGS98 participants (response rate 62%). The participants were interviewed (health questionnaire, nutrition questionnaire, medical interview, medicines interview) and examined (including laboratory analyses of biomarkers). For the trend analyses additional data from the health survey OW91 [21] as well as BGS98 [22] were used. For reasons of comparability only the age groups 25-69 years were included into the analyses. The evaluations covered datasets of 7,463 persons from OW91, 5,684 persons from BGS98 and 5,305 persons from DEGS1.

### 2.3 Estimate of the consumption quantity of pure alcohol

In the DEGS1 food frequency questionnaire the consumption frequencies and consumption quantities of altogether 53 food groups referred to the last four weeks before the survey were conducted. These include the alcoholic beverages beer, wine, high-proof beverages and cocktails and/or mixed beverages. In order to determine the frequency, the question asked was for instance “How often have you consumed wine, sparkling wine or fruit wine?”. The interviewees had the possibility to answer either “never”, “once a month”, “2-3 times a month”, “once to twice per week”, “3-4 times per week”, “5-6 times per week”, “once a day”, “twice a day”, “3 times a day”, “4-5 times a day” or “more often than 5 times a day”. The quantity consumed was determined with the question “If you drink wine, sparkling wine or fruit wine, how much do you drink in most cases?”. The possible answers were in this case “1 glass (125ml), 2 glasses, 3 glasses, 4 glasses, 5 glasses (or more)”. For the other beverages the possible answers for the quantity determination were different. Beer was, for instance, measured in 330ml bottles, cocktails/mixed drinks as number of beverages and high-proof beverages as glasses of 2cl.

The frequency and quantity data as well as the standard values for the mean alcohol contents of the beverages per litre – beer 38.11g, non-alcoholic beer 3.97g, wine 87.34g, distilled spirits 262.02g and cocktails/mixed drinks 75g – were used to estimate the average amount of alcohol in gram per day with the following formula:



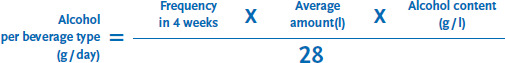



For the calculation of the indicator “harmful alcohol consumption” the daily drinking quantity of more than 10g pure alcohol for women and 20g pure alcohol for men was classified as harmful (see Infobox Forms of alcohol consumption harmful to health).

### 2.4 Further variables

The health behaviour is frequently conditioned by factors of different dimensions. This is why in this contextual analysis not only age and the socio-economic status but also the characteristics health condition (subjective assessment), health behaviour (tobacco consumption, sports activities) and social support have been taken into consideration.

For the determination of the socio-economic status an index was calculated by taking into account the three status dimensions education, occupation and income [23]. The self-rated health condition was surveyed on the basis of a question from the Minimum European Health Module (MEHM): “How is your health in general?” [24]. The five possible replies were subsequently summed-up in the two categories “very good/good” and “fair/bad” (including “very bad”).

“Smokers” were defined in this analysis as persons who stated that they smoke on a daily basis or occasionally.

The sports activity was recorded by asking about the frequency of sports activities during the past three months [25]. If a person selected the category “no sports activities”, this person was considered to be inactive in terms of sports and when selecting one of the other four categories (from “below one hour per week” to “four hours per week and more”), the person was considered as active in terms of sports activities.

The social support was measured based on the “Oslo-3 Items Support Scale” (Oslo-3) [26]. The three questions of the tool refer to the number of persons on whom one can count if one has serious problems, the assessment of the concern that people show in what one is doing as well as the possibility to get practical help from neighbours. The calculated overall score was sub-divided into the three categories “poor support”, “moderate support” and “strong support” [27].

### 2.5 Statistical analysis

The cross-sectional and trend analyses were conducted with a weighting factor which corrects deviations of the sample from the population structure (status: 31.12.2010) in terms of age, gender, region and nationality as well as type of community and education. All analyses were based on the survey procedures of Stata SE 14 taking into account the weighting and cluster design effect. The descriptive analysis of harmful alcohol consumption, differentiated by further variables (gender, age, socio-economic status, surveying period), was made by calculating prevalences with 95% confidence intervals. Based on the Pearson χ^2^ test the differences in harmful alcohol consumption between groups (e.g. men and women) as well as between the survey periods were checked for statistical significance (p<0.05). In sub-groups the 95% confidence intervals were used in order to identify significant differences (e.g. differences between two periods of time in a certain age group of men). In order to adjust the effects of important factors which are associated with harmful alcohol consumption against each another, multivariate analyses (binary logistic regressions) were calculated separately by gender. The dependent variable represented harmful alcohol consumption (reference group: no harmful alcohol consumption) and the independent variables were the age, the socio-economic status, the subjectively assessed health condition, the smoker status, the sports activities as well as social support.

## 3. Results

### 3.1 Frequency of harmful alcohol consumption and multivariate analysis

**Fig. 1:**
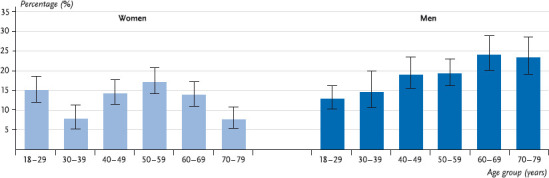
Prevalence of harmful alcohol consumption by age (n = 7,006) Source: DEGS 1 2008 – 2011

The results of DEGS1 show that 13.1% of the women and 18.5% of the men consume on a daily average more than 10g (women) or 20g pure alcohol (men) and hence tend to show harmful consumption. Men consume significantly more frequently alcohol in harmful quantities than women. The share of men with harmful alcohol consumption rises with age and reaches the maximum in the age group 60 to 69 years. In this age group almost one-fourth of the men shows harmful alcohol consumption. For women the lowest prevalence of harmful alcohol consumption is amongst the 30 to 39-year-olds and the highest amongst the 50 to 59-year-olds (Figure 1).

**Fig. 2:**
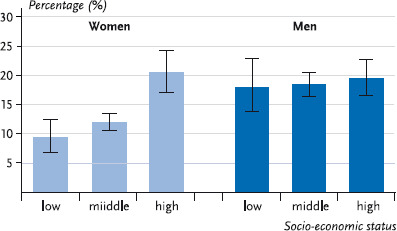
Prevalence of harmful alcohol consumption by socio-economic status (n = 6,966) Source: DEGS 1 2008 – 2011

In addition to the differences in prevalence by age, women show significant differences in harmful consumption by socio-economic status: the prevalence of harmful consumption is significantly higher for women with a high socio-economic status than for women from medium and low status groups. For men there are no significant differences in this respect (Figure 2).

A multivariate analysis of correlations between harmful alcohol consumption and socio-demographic factors, health condition, health behaviour as well as social support shows that the factors associated with harmful alcohol consumption are partly different between women and men (Table 1). Whereas a harmful alcohol consumption is related to age, the socio-economic status and tobacco consumption for women, there is, by contrast, no connection to the socio-economic status for men. Unlike women, harmful alcohol consumption for men is, however, associated with a better self-assessed health condition. In concrete terms, the frequency of harmful alcohol consumption is lower in women between the age of 30 and 39 than for younger women. For the other age groups there is, however, no significant correlation between age and alcohol consumption. Moreover, women with a low and medium socio-economic status consume less frequently harmful quantities of alcohol than women from the high status group. The correlations between harmful alcohol consumption, age and the socio-economic status of women from the bivariate analysis can be confirmed taking into account additional factors. Furthermore, female smokers have more frequently a harmful alcohol consumption than female non-smokers. As far as men are concerned, there is likewise a correlation between harmful alcohol consumption and age, taking into account additional factors, but there is a different pattern than for women. The frequency of harmful alcohol consumption rises for men continuously with age and is for the age group 70 to 79 years three times as high as for the youngest age group. Moreover, men who assess their health condition as very good or good consume more often harmful quantities of alcohol than men who assess their heath condition as moderate or poor. In a similar way as for women, smokers have twice as frequently a harmful alcohol consumption than non-smokers.

### 3.2 Trends in harmful alcohol consumption

**Fig. 3:**
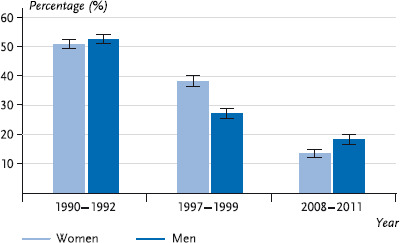
Trend of harmful alcohol consumption for women and men (25 – 69 years; n = 18,452) Source: OW91 (1990 – 1992), BGS98 (1997 – 1999), DEGS1 (2008 – 2011)

Since in earlier surveys by RKI (see 2.2 Method) data on frequency and quantity of alcohol consumption had been polled, it is also possible to present the development of harmful alcohol consumption from 1991-2011 for the age groups of 25-69-year-olds over time. This shows both for men and for women a strong decline in harmful alcohol consumption across the entire surveying period. Whereas in 1990-1992 every second woman and every second man consumed harmful quantities of alcohol, this was only the case for every seventh woman and every fifth man in 2008-2011 (Figure 3).

**Fig. 4:**
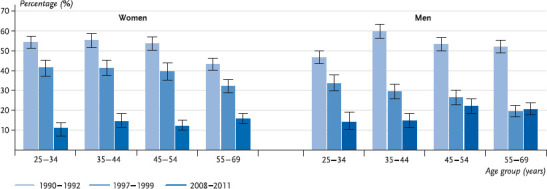
Trend of harmful alcohol consumption for women and men (25-69 years) by age groups (n = 18,452) Source: OW91 (1990 – 1992), BGS98 (1997 – 1999), DEGS 1 (2008 – 2011)

The analysis by age shows for women a constant decline in harmful alcohol consumption across all age groups. For men this trend is confirmed for those aged between 25 and 34 and those aged between 35 and 44 years. For men in the higher age groups (45-54 and 55-69 years) it has likewise been possible to establish a decline in risky alcohol consumption from 1990-1992 versus 1997-1999. In the period from 1997-1999 versus 2008-2011 there is, however, only a slightly decline for men aged 45-54 years, whereas men aged 55-69 years showed a stagnating share of consumers with harmful quantities between the two periods (Figure 4).

## 4. Discussion

13.1% of the women and 18.5% of the men aged between 18 and 79 years consume on average more than 10g (women) or 20g pure alcohol (men) per day and therefore tend to have a harmful consumption. At the interpretation of the data it has to be taken into account that in the event of self-reporting of alcohol consumption there can be an underestimation because the interviewees tend frequently towards a socially desired answering behaviour in view of their actual drinking behaviour. Nonetheless all representative German data sources show prevalences of harmful alcohol drinking quantities on a comparable level. The results of DEGS1 are, for instance, comparable to those from the epidemiological survey (ESA) 2012. Amongst the interviewees of this study 12.8% of the women and 15.6% of the men aged between 18 and 64 years consume alcohol in harmful quantities (28). As far as this comparison is concerned, it must be taken into account that the limit value for harmful consumption is 12g (women) or 24g (men) pure alcohol per day in the ESA and that only the age groups between 18 and 64 years are covered. The results from DEGS1 can be considered as robust against this backdrop.

Moreover, the DEGS1 data show that among men the consumption prevalences are highest in the age groups from 60 to 69 years and from 70 to 79 years. The age group of men aged 18 to 29 years shows, by contrast, comparatively low prevalences. As far as this is concerned, the DEGS data are different from those of other German study results. The data from the alcohol survey 2012 of BZgA for the age group of men aged between 18 and 25 years show values of 19.2% for the harmful consumption [29]. The low prevalences of women aged between 30 and 39 years are matching in DEGS1 and ESA and are probably, in addition to pregnancy and breastfeeding, due to the lower alcohol consumption of mothers [30]. In conformity with all other studies, DEGS1 shows generally higher shares of men with harmful consumption compared to women. This gender difference is explained, amongst others, by a different habitual drinking behaviour of women and stronger social sanctions against women in the event of deviating behaviour [31].

The prevalence of harmful consumption in accordance with AUDIT-C is with 25.6% for women and 41.6% for men significantly higher than the prevalence using the quantity frequency index [32]. In this connection it has to be taken into account that the AUDIT-C can also be applied together with the limit values used in DEGS1 for the screening of abusive and dependent alcohol consumption and moreover contains information about heavy episodic drinking [33]. Furthermore, it does not refer to the consumption of the past four weeks but asks for drinking behaviour in general.

The stratification according to the socio-economic status shows a gradient for women to the effect that in the high status group the highest prevalence of harmful consumption can be found. For men this cannot be observed. A comparable result was already obvious in the evaluations of BGS98 [34]. In addition, international studies confirm that women with a higher educational level are more likely to drink harmful amounts of alcohol than women with a lower educational level, while this correlation does not exist for men [35, 36]. Women with a higher socio-economic status possibly orient themselves less towards the traditional role sort compared to women from lower status groups. Analyses from countries with low or medium income suggest that there is in particular a correlation between paid employment of women and harmful alcohol consumption [37].

The effects of harmful alcohol drinking quantities are, however, not equally serious in all status groups: international studies show that the same high alcohol consumption causes more damage to health in disadvantaged groups than in privileged groups. This “alcohol harm paradox” is explained, amongst others, by the fact that in disadvantaged groups health risks such as smoking, obesity, poor nutrition and lack of physical activity are more often present in combination and the persons concerned, moreover, have a higher prevalence as far as heavy episodic drinking is concerned [38]. Together these factors increase the risk of diseases and alcohol-related damages such as accidents and injuries. A meta-analysis including data from 25 countries shows that women and men with a lower educational level have a higher risk concerning alcohol-related damages, even when taking into account the different drinking patterns. The fact that problems become significantly more visible in persons with a low educational level than in persons with a higher level, is attributed to different social and environmental resources in coping with stress or other problems [39].

The results of the regression analysis suggest, however, that men who assess their health condition as very good to good drink to a higher extent alcohol in harmful quantities regardless of their age, socio-economic status and other health behaviour. There is a tendency to see this correlation also in women. Persons who perceive their health condition as very good to good probably take care less frequently of a moderate alcohol consumption compared to persons who perceive themselves as impaired in terms of health. A positive correlation between the self-assessed health condition and harmful alcohol consumption was also confirmed in other studies [40].

Moreover, the results of the regression analysis prove the correlation between smoking and alcohol consumption, regardless of age, socio-economic status and health condition. Two approaches seem appropriate to explain this correlation: on the one hand, smokers seem to be more disposed to consume alcohol also in higher amounts. On the other hand, situations in which alcohol is consumed enable in most cases also to smoke on that occasion [41]. Since a smoking ban in bars and pubs has so far not been comprehensively introduced, this correlation continues to apply in Germany. Not least because alcohol and tobacco consumption increase together the risk of subsequent morbidity and mortality [42, 43], health promotion and prevention measures should not address an individual health behaviour in isolation, but should focus altogether on promoting a healthy lifestyle.

The results of the trend analysis prove a strong decline in consumption of harmful alcohol drinking quantities between 1990 and 1992 as well as 2008 and 2011, for men from 52.6% to 18.3%, for women from 50.9% to 13.6% (referred to the 25 to 69-year-old population). By analogy to DEGS1, the trend analyses of the ESA data likewise prove a decline in harmful consumption (age group 18 to 59 years) between 1995 and 2012 [14]. Finally, the regularly collected values of the alcohol survey of BZgA show that during the past years the prevalence of harmful alcohol consumption decreased for young men aged between 18 and 25 years. The share of 18 to 25-year-old women who drink harmful amounts of alcohol has again been increasing since 2012 [15].

Overall, the decline in alcohol consumption is not only revealed by population studies but also by consumption statistics: since 1991 the annual per capita consumption of the population aged 14+ decreased from 14.5 litres to 11.6 litres pure alcohol in 2014. This decrease is mainly attributable to a lower beer consumption. The consumption of wine and spirits has undergone only unessential changes [44].

Even if the trend analyses suggest altogether a declining alcohol consumption among the population, Germany ranges on an international comparison basis, measured in terms of per capita consumption of pure alcohol of the population aged ≥15, above the average of the EU Member States [2]. The growing rates of harmful alcohol consumption amongst young women [15] as well as the stagnating declines of harmful alcohol consumption among men aged 45 to 69 years are an indication of special target groups for health promotion and prevention. Moreover, it has to be taken into account that a considerable part of the persons consuming on average less than 10g (women) or 20g pure alcohol (men) per day can have problematic alcohol consumption, in particular heavy episodic drinking and not be reached by specific prevention measures. Apart from a consistent compliance with the statutory framework conditions, the adherence to the concept of situational abstinence during pregnancy and breastfeeding, at the workplace, in road traffic and during sports, additional options should be developed to reduce alcohol consumption on a social level. This includes a review of the possibilities in the field of pricing and regulations on the availability of alcohol, an increasing awareness of the problem on the political and community level as well as the promotion of a culture of “closely watching” and an alcohol-free or low hazard consumption behaviour in different life phases and life worlds. Finally, early detection and early intervention should be enhanced together with the support of addiction-burdened families and their children. At the same time an occasional glass of alcohol in general is not detrimental to health. In order to be able to achieve a reduction of alcohol consumption in the population, the propagation of responsible dealing with alcoholic beverages is a useful approach. For a low-risk enjoyment an orientation towards limit values which have been formulated for healthy adults makes sense [45]. As prevention messages these recommendations are part of the campaign “Know your limit” of the Federal Office for Health Education (see Infobox Low-risk alcohol consumption).

**Infobox: Low-risk alcohol consumption.**

**Enjoying responsibly, adhering to the limit**

8 tips for health-conscious alcohol consumption

1. As a woman you should not drink more than one standard glass of alcohol per day, as a man no more than 2 standard glasses per day.
2. Do completely without alcohol on at least two days per week.
3. Avoid to get drunk.
4. Do without alcohol at the workplace, in road traffic and at sports.
5. No alcohol during pregnancy and breastfeeding.
6. Do not serve any alcohol to children and check the alcohol consumption of adolescents.
7. Pay particular attention to your alcohol consumption as a senior person.
8. Avoid to combine alcohol and medicines and clarify when your health should prevent you from drinking alcohol.

http://www.kenn-dein-limit.de

**Figure:**
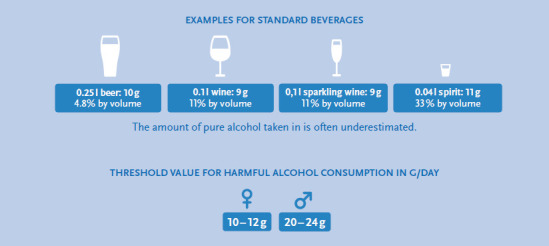


## Key statements

- 13% of the women and 19% of the men aged between 18 and 79 years drink alcohol in harmful quantities.
- Women with a high socio-economic status have a risk which is double as high to drink a harmful quantity of alcohol compared to women from medium and lower status groups.
- The share of consumers with harmful quantities increases in the event of men with age; for women the share is highest in the age group 50 to 59 years.
- The per capita consumption of pure alcohol of those aged ≥15 has likewise declined since 1990. Nonetheless it continues to be above the average of the EU Member States.

## Figures and Tables

**Table 1:** Correlation between harmful alcohol consumption and socio-demographic as well as health-related factors. Results of binary logistic regressions by gender (n = 6,757) Source: DEGS 1 2008 – 2011

	Women OR (95 %-CI)	Men OR (95 %-CI)
**Age** 18 – 29 years 30 – 39 years 40 – 49 years 50 – 59 years 60 – 69 years 70 – 79 years	Ref. **0,5 (0,3 – 0,8)** 1,0 (0,7 – 1,4) 1,3 (0,9 – 1,9) 1,2 (0,8 – 1,7) 0,7 (0,5 – 1,2)	Ref. 1,1 (0,7 – 1,8) **1,8 (1,2 – 2,6)** **1,8 (1,3 – 2,7)** **2,7 (1,9 – 3,9)** **3,1 (2,0 – 4,7)**
**Social status** Low Medium High	**0,5 (0,3 – 0,7)** **0,5 (0,4 – 0,7)** Ref.	0,8 (0,5 – 1,3) 0,9 (0,7 – 1,1) Ref.
**Subjective health condition** Very good/good Fair/bad	1,4 (1,0 –2,0) Ref.	**1,5 (1,2 – 2,0)** Ref.
**Smoking** Yes No	**1,7 (1,3 – 2,2)** Ref.	**2,0 (1,5 – 2,6)** Ref.
**Sports activities** Yes No	1,3 (1,0 – 1,7) Ref.	0,8 (0,6 – 1,1) Ref.
**Social support** Poor Moderate Strong	1,3 (0,8 – 2,0) 1,0 (0,8 – 1,3) Ref.	0,7 (0,5 – 1,0) 1,0 (0,8 – 1,3) Ref.

OR = Odds ratios; 95%-CI = Confidence interval; Ref. = Reference group; Bold: significant (p<0.05)
